# Effects of Lumbar Stabilization Exercises on Isokinetic Strength and Muscle Tension in Sedentary Men

**DOI:** 10.3390/bioengineering10030342

**Published:** 2023-03-08

**Authors:** Seunghyeok Yeom, Hyeongdo Jeong, Hyungwoo Lee, Kyoungkyu Jeon

**Affiliations:** 1 Division of Sport Science, Incheon National University, 119 Academy-ro, Yeonsu-gu, Incheon 22012, Republic of Korea; 2 Functional Rehabilitation Biomechanics Laboratory, Incheon National University, 119 Academy-ro, Yeonsu-gu, Incheon 22012, Republic of Korea; 3 Department of Human Movement Science, Incheon National University, 119 Academy-ro, Yeonsu-gu, Incheon 22012, Republic of Korea; 4 Sport Science Institute, Incheon National University, 119 Academy-ro, Yeonsu-gu, Incheon 22012, Republic of Korea; 5 Health Promotion Center, Incheon National University, 119 Academy-ro, Yeonsu-gu, Incheon 22012, Republic of Korea

**Keywords:** lumbar stabilization, erector spinae muscle, tensiomyography, isokinetic, strength

## Abstract

Lumbar stabilization exercises (LSE) lead to high levels of erector spinae muscle (ESM) activation, which has a positive effect on improving physical function. The purpose of this study is to identify factors explaining changes in muscle strength after 7 weeks of LSE and to evaluate changes in stiffness and contraction of the ESM. All participants (male: n = 42, age = 28.26 ± 10.97) were assessed for 60°/s isokinetic extensor muscle strength and tension using a tensiomyography (TMG) and isokinetic device before and after LSE. Maximum displacement (Dm) and average velocity up to 90% Dm (Vc 90) were significantly different before and after LSE. Additionally, participants’ 60°/s isokinetic extensor strength was significantly higher after exercise. A regression analysis was conducted to test the explanatory power of the variables, and positive results were obtained in the increase in extensor strength before and after Vc 90 and LSE. Furthermore, statistical significance was set at *p* < 0.05. After LSE, the increase in 60°/s isokinetic extensor strength and ESM’s Dm and Vc 90 can be interpreted as positive changes post-exercise in endurance muscles with a higher percentage of type I fibers. Our results can contribute to predicting the long-term exercise effect in sedentary workers and developing an individualized strategic exercise program.

## 1. Introduction

The World Health Organization (WHO) recommends that adults participate in at least 150–300 min weekly of moderate to intense physical activity to maintain good health [1]. Meanwhile, the COVID-19 pandemic has caused many changes, such as in physical activity and sedentary lifestyles, in our daily lives [2]. Especially, most people worldwide have been reported to show an insufficient level of physical activity and increasingly sedentary lifestyles [3]. A decrease in physical activity causes muscle imbalance and a decrease in flexibility, which cause chronic musculoskeletal disorders [4]. The lack of physical activity due to long hours of sedentary work could cause musculoskeletal pain due to the weakening of muscles required for core and lumbar stabilization or overpressure on the lumbar area [5,6,7,8]. Such dysfunction of the lumbar muscles may, in turn, induce lumbar pain [9,10].

Notably, as the primary muscle responsible for lumbar stabilization, the erector spinae muscle (ESM) is known to provide direct protection and support to the spine; the basic bearing required for static and dynamic stability during walking, running, and maintaining posture is given by the ESM [11,12]. The reduced physical functions due to insufficient physical activities can be improved through lumbar stabilization exercises (LSE), and such exercises as the curl-up, bird-dog, bridge, and side-crunch are effective in improving physical functions based on the high-level activity of the lumbar erector spinae (LS) muscles [13,14,15]. Previous studies reported that treatment with at least 4–6 weeks of LSE effectively improved participants’ LS muscle functions and sense of balance [16,17,18]. A common exercise aimed at preventing low back pain and improving the function of certain trunk muscles is used as a lumbar stabilization exercise [19].

Tensiomyography (TMG) is a non-invasive method to measure muscle rigidity and fatigue in a static posture. The muscle state changes are measured by assessing the displacement and time of muscle contraction induced by 1 ms of electrical stimulation (0–100 mA) to a single muscle fiber bundle [20,21]. The method was also reported as a valid, non-invasive method to measure muscle fiber composition [22]. Additionally, TMG is not only less sensitive to external noise, but it is also not affected by skin resistance or sweating [23].

For the ESM mediating the LS, numerous studies have reported the positive effects of long-term LSE in improving LS muscle functions [11,16,17,18,24]. However, few studies have investigated the changes in single muscle fibers that constitute the LS muscles following an exercise. While TMG allows fluent measurements, there is a lack of studies analyzing the factors accounting for the variation in muscle strength in TMG. Moreover, no study has assessed the variation in ESM rigidity and contraction properties using TMG after a long-term exercise with a simultaneous focus on the correlation with muscle strength measured via the changes in the isokinetic muscle strength. This study thus examined the TMG variables that can explain the variation in muscle strength based on the changes in ESM strength and TMG variables after 7 weeks of LSE in sedentary male office workers. The purpose of the study was to identify the correlation between the LSE-induced changes in muscle tension and lumbar extensor muscle strength and to propose basic data for facilitating the development of a suitable and effective exercise program for lumbar stabilization in sedentary workers through the use of TMG.

## 2. Materials and Methods

### 2.1. Participants

This study was a single-group cross-over design. For this study, we included 50 sedentary adult male workers aged 20–40 years with a lower level of physical activity than the WHO recommendation. Exclusion criteria were participants with a history of musculoskeletal surgery within the past 3 months, back pain during physical activity, neurologic motor weakness, deformity (scoliosis), history of recent lumbar or abdominal surgery, systemic inflammatory disease, or psychiatric disease. Eight of them dropped out of the study because they missed either the exercise program or the test, leaving 42 individuals who had completed every session of the exercise program and the associated tests. This study was approved by the Institutional Review Board of the Incheon National University (INUIRB No. 7007971-202012-003A). The participants provided informed consent after receiving sufficient explanations regarding the study’s contents and procedures. Table 1 presents the physical characteristics of the participants.

### 2.2. Procedures

**Figure 1:**
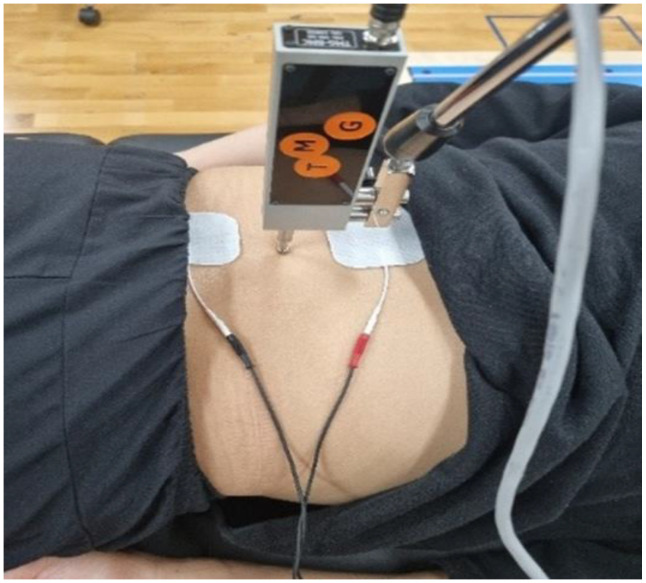
Measurement position of TMG.

After the enrollment, a pre-test was performed as the participants visited the lab before the exercise treatment. The TMG (TMG-100 System Electrostimulator, Ljubljana, Slovenia), which is a device used to analyze the contractile properties of muscles, was used to assess the mechanical and neuromuscular properties of the ESM. Caffeine intake may affect muscle contraction time and displacement [25]. So, the participants were requested to refrain from caffeine intake for 24 h before the measurement. Moreover, myofascial treatment and exercise that can cause muscle fatigue were restricted 48 h before measurement [26]. To apply the TMG to the participants’ ESM, they were guided to lie face down on the bed and position their legs at 5° flexion using a cushion, and wedge cushions were placed on the ankle and the anterior superior iliac spine (ASIS) to ensure minimal lumbar lordosis [27]. For accurate measurements, the TMG sensor (GK 40, Panoptik d.o.o., Ljubliana, Slovenia) was perpendicularly placed on a point in the thickest muscle belly marked by a pen upon the ESM’s isometric contraction on the same level as the iliac crest. Two electrodes (50 × 50, T.Y. Sherry International Co., LtD., Taiwan) were placed 2.5 cm from the sensor on either side (Figure 1) [28].

**Figure 2:**
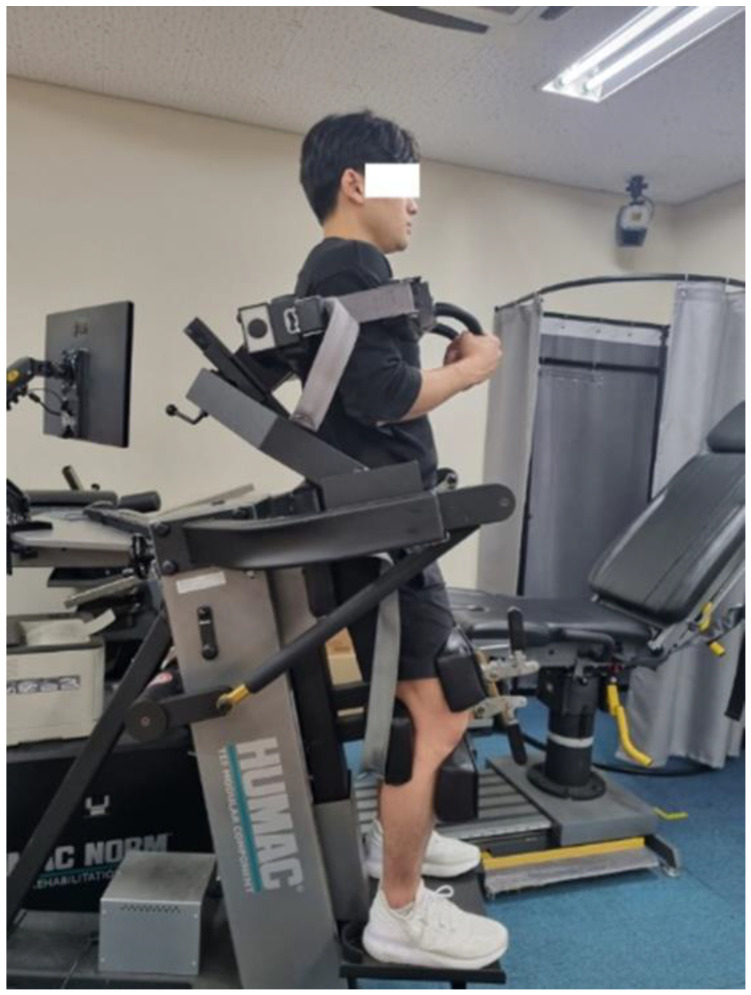
Measurement posture of trunk isokinetic strength.

For muscle measurements, electrical stimulation was first applied at 20 mA and increased by 20 mA until the maximal (100 mA) contraction displacement. Over 10 s of rest was allowed between each stimulus [21,29]. Two investigators took the measurements, one of whom was responsible for positioning the TMG on the ESM while the other performed the measurement program. All measurements were conducted from right to left. Following the TMG measurements, the maximum isokinetic muscle strength of the participant’s torso was measured using an isokinetic device (Humac Norm Testing & Rehabilitation, CSMi Medical & Solutions, Stoughton, WI, USA), and to prevent injury, the participant was guided to perform a 10-min warm-up such as dynamic stretching trunk flexion, extension, hip hinge, leg extension, and flexion, and perform the main motions five times at 60°/s (Figure 2) [30].

After the pre-test, the participants underwent 7 weeks of LSE, whose intensity gradually increased. Based on Borg’s CR-10 scale, the exercise proceeded for 55 min per session, three times a week, at a level of 5–8 RPE (rated perceived exertion) [31]. The exercise program was designed to be specialized for core strengthening in accordance with previous studies and consisted of a 10 min warm-up through dynamic stretching, 40 min of main exercise, and a 5 min cool-down through post-exercise stretching [15,32]. The main exercise focused on core muscles such as the lower back, abdominals, and hips that contribute to core stabilization for improved trunk muscle strength and stability. 1–3 weeks core exercise program comprised 5 sets of 10 reps with 5 s of contraction and 10 s of relaxation, and 5 sets of 10 reps with isometric contraction during 10 s. A 4–7 week core exercise program comprised 5 sets of 15 reps with 5 s of contraction and 10 s of relaxation, and 5 sets of 15 reps with isometric contraction during 10 s. The core stabilization exercise program of this study was conducted by a total of 4 people: 2 main instructors and 2 auxiliary instructors. Adherence and compliance for this program were used as the basis for direct observation by the instructors in charge of conducting and supervising (Table 2).

### 2.3. Isokinetic Muscle Strength Test

In order to measure the ESM’s strength, the isokinetic muscle strength measuring device (Humac Norm Testing and Rehabilitation, CSMi Medical & Solution, Stoughton, MA, USA) was used. The measured variables of trunk extensors using this equipment showed high reliability [33]. The participants were guided to perform a 10 min warm-up, such as dynamic stretching for trunk flexion, extension, hip hinge, leg extension, and flexion, to prevent injury, and were given adequate explanation and practice time on each motion before the measurements. To assess the maximal muscle strength during the trunk flexion-extension motion, the motion was performed five times at 60°/s [30]. The participants were fixed with a pad to fix the trunk, thigh, and pelvis during the measurements for accurate measurement. To prevent the torso from swaying, a pad for the upper body was attached at the inferior angle of the scapula. The isokinetic device’s dynamometer was aligned to the top of the iliac crest as the motional axis of the body, and taking the body’s vertical position as 0°, the range of motion was set at 15° of extension and 90° of flexion. The mean of five trials of absolute muscle strength variables was divided by the body weight of each participant to derive relative muscle strength variables.

### 2.4. Tensiomyography

**Figure 3:**
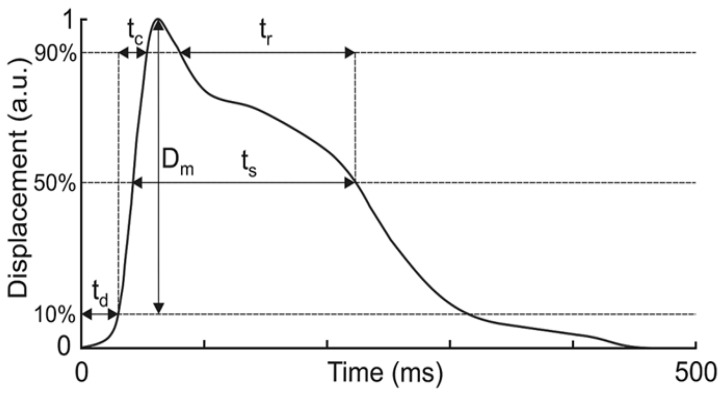
Variables of Tensiomyography measurement (International society of tensiomyography).

In this study, TMG was used to analyze the contractile properties of muscles and was used to assess the mechanical and neuromuscular properties of the ESM, using a TMG sensor with a 0.17 N/mm spring constant [27]. The TMG involved a 1 ms electrical stimulus (0~100 mA) to elicit the muscle contraction reaction, while the following variables were measured: the maximal displacement (Dm), the time taken to reach 10–90% of Dm (contraction time [Tc]), the time between the initiation of stimulus and 10% of Dm (delay time [Td]), the time of retention between 50% of Dm and 50% of the falling contour (sustain time [Ts]), and the time taken to reach 90–50% of Dm from the falling contour (relaxation time [Tr]) [34] (Figure 3).

The TMG muscle measurement is reported to show the intra-class correlation coefficient as follows: Tc: 0.62–0.98, Ts: 0.71–0.96, Tr: 0.62–0.96, Td: 0.47–0.98, Dm: 0.86–0.99, and the mean velocity until 90% Dm (Vc 90): 0.93–0.99. The reliability of TMG was either good or very good, scoring 0.9 ≤ 1 [35,36]. Nonetheless, the coefficient of variation (CV) was poor for Tr and Ts. The reliability was determined to be acceptable at CV < 10% but compared to the commonly used variables (Tc [2.6–9.4%], Td [1.16–4.2%], and Dm [8.0–14.8%]), Ts (5.3–21.3%), and Tr (6.4–32.8%), there was a large variation among the measured values so that they were excluded from this study [35]. In addition, as Tc is the time taken to reach 10–90% of Dm and Td is the time taken to reach 10% of Dm, they were influenced by the level of Dm. To complement this, the method of calculating the contraction velocity for the muscle, Vc 90, was calculated as shown in the equation below [37]. All right- and left-side values of all variables were summed, and the mean values were calculated and analyzed using the ESM.

(1) $$\mathrm{Vc}\;90=\frac{\left(\mathrm{Dm}\times 0.9\right)}{\left(\mathrm{Tc}+\mathrm{Td}\right)}$$

### 2.5. Statistical Analysis

All data were statistically analyzed using SPSS 26.0 for Windows (IBM, Chicago, IL, USA). The mean and standard deviation were calculated for all measured values. For the data normality test, the Shpiro–Wilk test was used, and normality was verified. A paired *t*-test was performed to identify the differences in the TMG variables and isokinetic muscle strength before and after LSE. Further, a multiple linear regression analysis was performed to test the explanatory power between isokinetic extensor muscle strength and TMG variables both before and after the application of the exercise program. The level of significance was set at *p* < 0.05.

## 3. Results

### 3.1. Isokinetic Extensor Muscle Strength & TMG

The TMG and 60°/s isokinetic extensor muscle strength showed significant differences after the completion of 7 weeks of core exercises. Tc and Td showed no significant difference, whereas Dm and Vc 90 showed a significant increase after the completion of a 7-weeks core exercise program. The isokinetic extensor muscle strength also showed a significant increase (*t*_41_ = −5.637; *p* = 0.000; Cohen d = 0.58; 95% CI = −63.55 to −30.02). Our results showed a significant increase in the Dm (t_41_ = −3.236; *p* = 0.002; cohen’s d = 0.31; 95% CI = −1.13 to −0.26) and Vc90 *(t*_41_ = −3.842; *p* = 0.000; cohen’s d = 0.33; 95% CI = −0.03 to −0.01) a slight decrease in the Tc *(t*_41_ = 0.492; *p* = 0.625; cohen’s d = 0.09; 95% CI = −0.72 to 1.18) and Td *(t*_41_ = 1.593; *p* = 0.119; cohen’s d = 0.33; 95% CI = −0.54 to 4.59) without statistical significance (Table 3).

### 3.2. Regression Analysis

**Figure 4:**
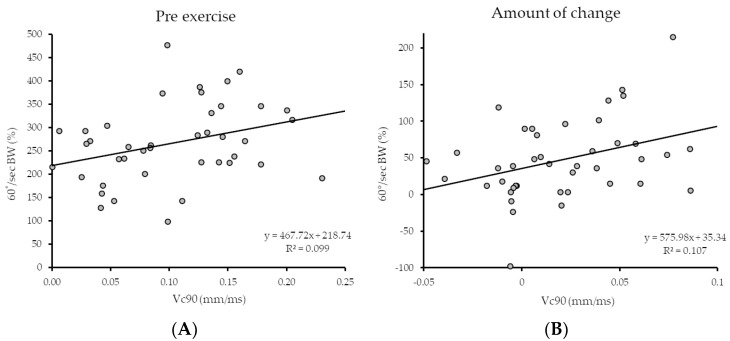
(**A**) Results of linear regression analysis of pre-exercise, (**B**) Results of linear regression analysis of Amount of change.

A regression analysis was performed to test the explanatory power of the TMG variables and isokinetic extensor muscle strength; among the TMG variables, Vc 90 had significant explanatory power. Positive results were also obtained for the initial extensor muscle strength and Vc 90 (Figure 4A) and the post-exercise increase in extensor muscle strength and Vc 90 (Figure 4B).

## 4. Discussion

The purpose of this single-group cross-over design study is to propose basic data for facilitating the development of a suitable and effective exercise program for lumbar stabilization in sedentary workers through the use of TMG and isokinetic muscle strength measuring devices. The effects of long-term LSE in improving LS muscle functions have been reported in numerous previous studies, but only a few have investigated the effects on the ESM with a role in lumbar stabilization. To determine the functional changes in the ESM after the 7-weeks LSE program, this study investigated the variation in isokinetic muscle strength as a TMG variable before and after the core exercise to verify the explanatory power of TMG variables and performance. The results showed that the lumbar extensor muscle strength significantly increased after the 7-weeks LSE exercise program, and the Dm and Vc 90 among the TMG variables also significantly increased after the 7-weeks LSE exercise program but not Tc or Td (Table 3). In addition, among the TMG variables, the Vc 90 was found to have a positive explanatory power for initial muscle strength and the variation in muscle strength (Figure 4).

Regarding the isokinetic extensor muscle strength of the trunk, there was a significant increase in relative extensor muscle strength at an angular velocity of 60°/s after the 7-weeks LSE exercise program rather than pre-isokinetic extensor muscle strength variables. According to a previous study, the isokinetic muscle strength was lower in the herniated lumbar disc group than in the healthy control group. The factors causing lumbar disease or dysfunction were overweight, smoking, and core overuse, and such lumbar problems were shown to be correlated with low isokinetic muscle strength [38,39]. A 6-weeks LSE study including sedentary adult women found a significant increase in 60°/s and 90°/s isokinetic muscle strength after exercise [40]. In another study, male adolescents aged 14–16 years performed LSE for 8 weeks, and their performance increased in the 20 m shuttle run test, 1 min push-ups, and curl-ups, except for aerobic exercises [41]. The lumbar extension exercise for 10 weeks was also reported to increase dead-lift strength and lumbar isometric extensor muscle strength [42]. As can be seen, performing LSE for 6 weeks or longer was reported in most studies to have improved core muscle strength and physical functions, with an association with enhanced lumbar functions. Therefore, our 7-weeks lumbar stabilization exercise program enhanced isokinetic extensor muscle strength of the trunk, which can enhance core stability and prevent musculoskeletal disorders caused by a lengthy sedentary lifestyle.

Regarding the mechanical and neuromuscular properties of the ESM, there was a significant increase in Dm and Vc90 after the 7-weeks LSE exercise program rather than the pre-mechanical and neuromuscular properties of the ESM in TMG variables except for Tc and Td. Among the TMG variables evaluated in this study, the Dm showed a significant increase after LSE. In contrast, a study conducted on adult men with 6 weeks of lower limb resistance training reported a fall in the Dm with hypertrophy of the rectus femoris but no change in the Dm with hypertrophy of the vastus lateralis after exercise [43]. However, in a previous study where male junior soccer players were trained through isometric-concentric contractions using six 5-s motions for 6 weeks, with much resemblance to this study, the Dm and performance increased significantly in the rectus femoris [44]. In addition, a study comparing the TMG variables according to muscle fiber variations by measuring the variables in the rectus femoris and the biceps femoris of strength sports (e.g., track and field) and endurance sports players showed that the latter players had higher Dm, Tc, and Td values [45]. This was attributed to the increase in Dm via adaptation to training in endurance sports players, with their muscles showing a higher proportion of Type I fibers. The ESMs examined in this study were high-endurance muscles with higher proportions of Type I fibers [46], and the results of this and previous studies collectively suggested that the ESM’s post-exercise Dm increase could be a positive response shown by muscles with higher proportions of Type I fibers. Furthermore, among the TMG variables, higher values of Td and Tc were correlated with higher positive values of the proportion of Type I fibers, suggesting that TMG may be a valid method of measuring the muscle fiber proportions [23]. The 7-week lumbar stabilization exercise program in this study was performed with isometric-concentric contractions similar to previous studies. As a result, it is suggested that Dm increased significantly after participating in the lumbar stabilization exercise program for 7 weeks, compared to before participating in the lumbar stabilization exercise program, as an adaptation to endurance training in ESM with many Type I fibers.

The 7-week lumbar stabilization exercise program in this study was performed with isometric-concentric contractions similar to previous studies. As a result, it is suggested that Dm increased significantly after participating in the lumbar stabilization exercise program for 7 weeks, compared to before participating in the lumbar stabilization exercise program, as an adaptation to endurance training in ESM with many Type I fibers.

The Vc 90 also showed a significant increase. This suggests a post-core exercise program increase in the contraction velocity of the erector spinae muscle. Our results showed a significant increase in the Dm (*p* = 0.002) and a slight decrease in the Tc (*p* = 0.625) and Td (*p* = 0.119) without statistical significance. The increase in the Vc 90 is thus presumed to be due to the significant increase in the Dm and the slight decrease in the Tc and Td. This was in agreement with a previous study reporting a fall in muscle contraction time and an improvement in jumping ability after 8 weeks of plyometric exercise [47]. In addition, muscle strength was enhanced after exercise, presumably due to the increase in the cross-sectional area (CSA) of Type II muscle fibers, motor unit recruitment, and motor unit firing frequency. Such enhanced muscle strength is likely to increase the muscle contraction velocity [48].

The regression analysis to examine the explanatory power of the TMG variables for the initial isokinetic extensor muscle strength and variation in muscle strength showed that, among the TMG variables, the Vc 90 had positive explanatory power for 60°/s extensor muscle strength, regarding the initial strength (R^2^_(abj)_ = 0.099, y=218.74+467.721x) (Figure 4A) and the variation (R^2^_(abj)_ = 0.107, y=35.338+575.98x) (Figure 4B). The results suggested that the Vc 90 was the most effective TMG variable for predicting pre-exercise extensor muscle strength and the post-exercise increase in extensor muscle strength. In line with this, a previous study reported a fall in the Vc ($\mathrm{Vc}=\frac{\mathrm{Dm}}{\mathrm{Tc}+\mathrm{Td}})$ concerning the reduced sprint time and thereby proposed it as an indicator of the contraction velocity to estimate the quantity of displacement per interval of time [49]. The measurement of maximum output for 30 s with 8% load using a cycle ergometer showed that the Vc was higher in the high-output group; therefore, it was proposed as a predictor of sports players’ agility [50]. In a study investigating athletes’ characteristics, agility-related performance was higher in short-distance runners with a higher proportion of Type II muscle fibers than in long-distance runners with a higher proportion of Type I muscle fibers, while the Tc, Td, and Dm were lower in short-distance runners [45]. Correlations between higher proportions of Type II fibers with lower Tc and Td, and between higher proportions of Type II fibers and higher muscle strength have been reported [22,51]. The high Vc 90 values in this study thus indicate relatively lower Tc and Td values, and the higher Vc 90 associated with higher initial muscle strength may imply a higher proportion of Type II muscle fibers. As Type II muscle fibers produce greater force and contraction velocity, the positive explanatory power of Vc 90 may be attributed to increased Vc 90 being correlated with increased initial muscle strength.

Furthermore, the increase in Vc 90 had positive explanatory power for the increase in muscle strength after exercise. This is presumably due to the increased contraction velocity and muscle strength after training with regard to the increased Type II muscle fiber activity and CSA [48,52]. In a previous study, an 8-week plyometric training program resulted in reduced contraction time and improved vertical jumping ability with increased CSA of Type II muscle fibers caused by exercise [47]. In this study, the increase in Vc 90 led to reduced contraction times, which decreased as the proportion of Type II muscle fibers increased [22]. The participants exhibiting a relatively high margin of increased muscle strength showed a greater increase in Vc 90, which may be due to the greater increase in the CSA of Type II muscle fibers after exercise, leading to a greater increase in muscle strength and contraction velocity.

The limitations of this study were that there was no control group and only healthy, young (20–40 year old) male subjects were recruited. Therefore, the results of this study are probably hard to apply to females, middle-aged or older populations, and also the population with higher physical activity. In future studies, it is necessary to further study the effect of the core stabilization exercise program by composing a control group and a female, middle-aged, or older population group.

## 5. Conclusions

This study was conducted to provide evidence of an effective LSE program by assessing the changes in the ESM’s mechanical and neuromuscular properties and isokinetic muscle strength before and after the 7-week LSE program in sedentary workers. After 7 weeks of LSE, the ESM’s Dm and Vc 90 showed significant increases, which may be a positive change such as the increase in the proportion of Type I fibers and the cross-sectional area (CSA) of Type II muscle fibers, as well as motor unit recruitment and motor unit firing induced by the 7-weeks LSE program. In addition, among the TMG variables, Vc 90 accounts for the initial muscle strength and the post-exercise increase in muscle strength, while TMG is likely to prove useful in predicting exercise’s effects in sedentary workers. Based on these findings, the prediction data of exercise’s effects on sedentary workers with low physical activity levels may lead to the development and modification of individually tailored strategic exercise programs. Future studies should include a control group and recruit participants from a female, middle-aged, or older population group to investigate the effects of a sedentary lifestyle and core stabilization exercise.

## Figures and Tables

**Table 1:** Physical characteristics of the participants.

Variables		Value
Participants (n = 42)	Age (years)	28.26 ± 10.97
	Height (cm)	174.78 ± 5.32
	Weight (kg)	73.57 ± 7.75
Physical activity	Vigorous-intensity (day/week)	0.27 ± 0.63
	Vigorous-intensity (min/day)	9.76 ± 21.15
	Moderate-intensity (day/week)	0.59 ± 0.92
	Moderate-intensity (min/day)	18.29 ± 32.40

Note. Data are mean ± standard deviation.

**Table 2:** 7 weeks core exercise program.

Practice Weeks	Exercises	Volume	Rest
1–3 weeks	Warm-up exercises	10 min	60 s between sets
	Abdominal crunch	5-s contraction	
	Back extension	10-s relaxation	
	Back bridge (right leg lift)	<10 reps, 5 sets>	
	Back bridge (left leg lift)	Isometric contraction	
	Side bridge (right side)	10-s hold,	
	Side bridge (right side)	<10 reps, 5 sets>	
	Cool-down exercises	5 min	
4–7 weeks	Warm-up exercises	10 min	60 s between sets
	Abdominal crunch	5-s contraction	
	Back extension	10-s relaxation	
	Back bridge (right leg lift)	<15 reps, 5 sets>	
	Back bridge (left leg lift)	Isometric contraction	
	Side bridge (right side, left leg lift)	10-s hold	
	Side bridge (right side, right leg lift)	<15 reps, 5 sets>	
	Cool-down exercises	5 min	

Note. Warm-up: whole body dynamic stretching, Cool-down: whole body static stretching.

**Table 3:** Results of TMG and isokinetic strength variables of the participants.

Variables		Pre	Post	*T*	*p*	Cohen’s *d*	95% CI Lower	95% CI Upper
Isokinetic strength (Extension)	60°/s BW (%)	268.81 ± 83.62	315.60 ± 76.31	−5.637	0.000 ***	0.58	−63.55	−30.02
TMG	Tc	15.16 ± 2.67	14.93 ± 2.34	0.492	0.625	0.09	−0.72	1.18
	Td	21.86 ± 8.35	19.84 ± 2.00	1.593	0.119	0.33	−0.54	4.59
	Dm	4.17 ± 2.41	4.87 ± 2.17	−3.236	0.002 *	0.31	−1.13	−0.26
	Vc 90	0.11 ± 0.06	0.13 ± 0.06	−3.842	0.000 ***	0.33	−0.03	−0.01

Note. Data are mean ± standard deviation. * *p* < 0.05, *** *p* < 0.001. Abbreviations: BW, Body weight; Tc, Contraction time; Td, Delay time; Dm, Maximum radial displacement; Vc90, Mean velocity until 90%; CI, Confidence interval.

## Data Availability

Data are not publicly available due to privacy.
